# Localized Proteomics of Amyloid Plaque Tissue in Down Syndrome and Alzheimer’s Disease

**DOI:** 10.1002/alz.088962

**Published:** 2025-01-03

**Authors:** Mitchell Marta Ariza, Dominique Leitner, Jianina Suazo, Ana Giusti Pedrosa, Evgeny Kanshin, Beatrix Ueberheide, Juan Fortea, Alberto Lleo, Thomas Wisniewski

**Affiliations:** ^1^ NYU Grossman School of Medicine, New York, NY USA; ^2^ Universitat Autònoma de Barcelona, Bellaterra, Barcelona Spain; ^3^ NYU, New York, NY USA; ^4^ New York University, New York, NY USA; ^5^ Sant Pau Memory Unit, Hospital de la Santa Creu i Sant Pau, Biomedical Research Institute Sant Pau, Universitat Autònoma de Barcelona, Barcelona Spain; ^6^ Centre of Biomedical Investigation Network for Neurodegenerative Diseases (CIBERNED), Madrid Spain; ^7^ Center for Biomedical Investigation Network for Neurodegenerative Diseases (CIBERNED), Madrid Spain

## Abstract

**Background:**

Down syndrome (DS) is strongly associated with Alzheimer’s disease (AD), attributable to *APP* overexpression, displaying common features with early‐onset AD (EOAD) and late‐onset AD (LOAD) like Amyloid‐β (Aβ) and tau pathology. Here, we evaluated the Aβ plaques proteome of DS, EOAD and LOAD.

**Method:**

We used unbiased localized proteomics to analyze amyloid plaques and the adjacent plaque‐devoid tissue (‘AD non‐plaque’) from post‐mortem paraffin‐embedded tissues in three subtypes of AD (n = 20/group): DS (59.8 ±4.99 y/o), EOAD (63 ±4.07 y/o), and LOAD (82.1 ±6.37 y/o). Functional associations were analyzed using Gene Ontology (GO) enrichment and protein interaction networks.

**Result:**

We identified 132, 192 and 128 differentially abundant proteins in DS, EOAD and LOAD Aβ plaques (FDR<5%, fold‐change<1.5). Common plaque‐associated proteins constituted 14% across all groups, with 15% only in DS, 32% in EOAD, and 17% in LOAD. Significant correlations (p<0.0001) existed between DS and EOAD (R2 = 0.77) and DS and LOAD (R2 = 0.73), compared to EOAD and LOAD (R2 = 0.67). Top BP GO terms (*p*<0.0001) were lysosomal transport, regulation of the immune system process and lysosome organization for DS, EOAD and LOAD, respectively. Protein networks revealed a characteristic signature across all AD subtypes involving APP metabolism, immune response, and lysosomal pathways. We identified 263, 269, and 301 differentially abundant proteins in DS, EOAD and LOAD non‐plaques. Common differentially abundant proteins constituted 12% across AD subtypes, with 24% only in DS, 14% in EOAD and 26% in LOAD. DS correlated significantly (*p*<0.0001) with EOAD (R^2^ = 0.59) and with LOAD (R^2^ = 0.33), compared to EOAD and LOAD (R^2^ = 0.79). The top BP GO term for all three subtypes was chromatin remodeling (*p* = 0.0013, *p* = 5.79 × 10^‐9^, and *p* = 1.69 × 10^‐10^, respectively). Additional GO terms for DS included extracellular matrix and integrin‐mediated signaling (*p* = 0.0068), while EOAD and LOAD were associated with protein‐DNA complexes and regulation of gene expression (*p*<0.0001).

**Conclusion:**

We identified proteome differences in Aβ plaques across AD subtypes. Correlation and functional analyses revealed that DS and LOAD plaque‐associated proteins primarily participate in lysosomal pathways, while EOAD proteins are linked to immune response. In contrast, EOAD and LOAD non‐plaque proteins correlated better compared to DS, with functional differences reflected in the top GO terms.

